# Paeoniflorin Enhances the Sensitivity of ER-Positive Breast Cancer Cells to Tamoxifen through Promoting Sirtuin 4

**DOI:** 10.1155/2022/6730559

**Published:** 2022-01-15

**Authors:** Pei Zhang, Nan Wu, Zhi-Jun Song, Zheng-Fu Tai

**Affiliations:** ^1^Yunnan Open University, Yunnan Vocational and Technical College of National Defense Industry, Kunming 650500, China; ^2^School of Medical, Yunnan University of Business Management, Kunming 650500, China; ^3^Guangxi Botanical Garden of Medicinal Plants, Nanning 530023, China; ^4^Sichuan Kelun Pharmaceutical Research Institute, Chengdu 610000, China

## Abstract

Tamoxifen is an effective drug for treating patients with advanced estrogen receptor-positive (ER+) breast cancer (BC), but not for all ER + BC patients. Drug tolerance is the biggest obstacle. In this study, we designed an experiment to investigate whether paeoniflorin affects the ER + BC cell's sensitivity to tamoxifen in the T47D and MCF-7 cell lines. Herein, we found that paeoniflorin inhibited cell proliferation without inducing apoptosis. However, it enhanced tamoxifen-induced apoptosis in both cell lines. Immunoblotting revealed that paeoniflorin significantly increased the already elevated Bax/Bcl2 protein expression ratio and the caspase 3 activity levels, both induced by tamoxifen. Paeoniflorin was also found to increase SIRT4 expression, and deletion of SIRT4 could significantly reverse the inhibition of cell proliferation induced by paeoniflorin and significantly decrease paeoniflorin-enhanced apoptosis induced by tamoxifen. Moreover, protein expression detection revealed that paeoniflorin enhanced the tamoxifen-induced inhibition of STAT3 activation. Besides, the deletion of SIRT4 could significantly increase STAT3 activation in the T47D and MCF-7 cells. In conclusion, paeoniflorin suppressed STAT3 activation to enhance the sensitivity of ER-positive breast cancer cells to tamoxifen through promoting SIRT4 expression.

## 1. Introduction

Endocrine therapy is an essential part of a comprehensive treatment for patients with estrogen receptor-positive (ER+) breast cancer (BC), including drugs that act as antagonists of the estrogen receptors, aromatase inhibitors, and drugs that promote the downregulation of the estrogen receptor [1, 2]. Tamoxifen (TAM) is the first-line drug for endocrine therapy for patients with ER + breast cancer. However, clinical data revealed that although the use of TAM in the early treatment period has apparent benefits for ER + breast cancer patients, some patients were prone to develop resistance to TAM and lead to tumor progression and metastasis [3, 4]. Statistics show that one-third of patients resistant to TAM are primary TAM resistant and 30–40% of patients who are sensitive to TAM in the early stage have secondary TAM resistance [3, 4]. Such long treatment with tamoxifen causes severe side effects and tamoxifen resistance, leading to breast cancer metastasis and even death [5, 6]. Thus, regulating the ER-positive breast cancer cell's sensitivity to tamoxifen can reduce its dosage and resistance.

Novel antitumor agents derived from natural sources have attracted exponential interest. The phytochemical compounds represent an alternative for the improvement of existing standard cancer therapies. Paeoniflorin is the main effective ingredient of the peony plant (*Paeonia* sp.). Its numerous pharmacological effects, including antitumor, anti-inflammatory, analgesic, hepatic and nerve protection, immune regulation, sedative, and hypnotic, are already described [7, 8]. Previous studies have found that paeoniflorin can inhibit the proliferation, metastasis, and apoptosis of several tumor cells [9, 10]. It can also regulate the multidrug resistance of gastric cancer cells and enhance sensitivity to chemotherapeutic drugs by inhibiting the NF-kB pathway activation [11, 12]. Meaningfully, paeoniflorin has been found to exert a tumor suppressor effect in breast cancer, including inhibiting the proliferation and metastasis of breast cancer cells by inhibiting the Notch-1 pathway [13]. Moreover, it can prevent hypoxia-induced epithelial-mesenchymal transition in human breast cancer cells through the PI3K/AKT pathway [14].

Sirtuin 4 (SIRT4), a member of the sirtuin proteins family, has been implicated in the regulation of cancer cell toxicity through the modulation of glutamate dehydrogenase [15] and enhancing the sensitivity of ER + BC cells to tamoxifen [16]. In this study, we investigate the paeoniflorin effects on the sensitivity of ER + BC cells to tamoxifen in the T47D and MCF-7 cells and explore its relationship with SIRT4.

## 2. Material and Methods

### 2.1. Cell Culture and Treatment

T47D (ATCC, CRL-3436) and MCF-7 (ATCC, CRL-3435) cells were cultured in DMEM medium (Gibco, 11965092) supplemented with 10% fetal bovine serum (Gibco, 16140071) at 37 °C with a 5% CO_2_ atmosphere. The T47D and MCF-7 cells were treated with different concentrations of paeoniflorin (sigma, 245-476-2) (0, 15, 30, and 60 *μ*mol/L) for different times (0, 24, 48, and 72 hours) or treated with different concentrations of tamoxifen (sigma, 234-118-0) (0, 1, 2, 4, 8, 16, 32, and 64 *μ*mol/L) for 48 hours.

### 2.2. Cell Viability Assay

1 × 10^4^ cells were seeded into 96-well cell culture plates. After stimulating with the different treatments, we removed the cell culture medium and washed the cells twice with PBS. Then, 100 *μ*L cell culture medium supplemented with 10 *μ*L CCK8 solution (Beyotime, C0038) was added to the cells and incubated for 2 hours. Lastly, we detected the OD450 and calculated the relative cell viability.

### 2.3. Apoptosis Assay

After stimulating with the different treatments, the cells were collected and washed twice with cold PBS buffer. Next, we detected the apoptosis of BMECs as described in the Annexin V-FITC/7-AAD apoptosis detection kit (A213-01, VAEYME, China). Briefly, 100 *μ*L of the binding buffer was used to resuspend an amount of 5 × 10^6^ cells, and then we added 5 *μ*L of Annexin V-FITC and 5 *μ*L of 7-AAD staining solution. After 15 minutes of incubation at room temperature in the dark, 400 *μ*L of binding buffer was added to the cell. The BMECs were analyzed in a flow cytometer.

### 2.4. SIRT4 Knockdown

A specific small interfering RNA for SIRT4 (si-SIRT4) was transfected into the T47D and MCF-7 cells to knock down the SIRT4 expression. 5 *μ*L of 50 nmol/L si-SIRT4 was mixed with 2.5 *μ*L Lipofectamine 2000 (Invitrogen, 11668019), and then 1 mL of Opti-MEM medium (Gibco, 31985088) was added to the mixture. After standing at room temperature for 20 minutes, the transfection mix was added to the cells. 4–6 hours later, we changed the medium to the complete cell culture medium. 48 hours after transfection, we proceeded with drug treatment. The siRNA sequences are described as follows: siNC: 5′-AACACAACUACUUGAGUAGCC-3′, si-SIRT4: 5′-UCCAAAGGGCCCCUUAUAGAU-3′.

### 2.5. Protein Expression Detection

The cells were harvested, and we extracted the total protein using a RIPA lysis buffer (Solarbio, R0010). After the detection of the protein concentration using a BCA kit (Solarbio, BC0020), 40 µg of the total protein was separated by 10% SDS-PAGE. Then, the protein was transferred to PVDF membrane and blocked with 5% nonfat milk at room temperature for 1 hour, and the primary antibody against SIRT4 (1 : 5000, Abcam, ab124521), Bax (1 : 1000, Abcam, ab32503), Bcl2 (1 : 1000, Abcam, ab182858), c-myc (1 : 1000, Abcam, ab32072), STAT3 (1 : 2000, Abcam, ab68153), and p-stat3 (1 : 1000, Abcam, ab267373) were incubated at 4 °C overnight. After that, we proceeded with the secondary antibody incubation at room temperature for 1 hour. The proteins were visualized with an ECL solution (WBKLS0100, Beijing Xinjingke Biotechnologies Co., Ltd, China) followed by densitometry analysis using ImageJ 3.0 (IBM, USA). *β*-actin was analyzed as a control.

### 2.6. Caspase 8 Activity Detection

We performed the caspase 8 activity detection assay using a kit (Beyotime, C1152) following the manufacturer's instructions. Briefly, we added 100 µL of lysate to every 2 × 10^7^ cells and then transferred the lysate to a 1.5 mL centrifuge tube and lysed for 15 minutes in an ice bath. Then, we collected the cell supernatant by centrifugation (20000g, 15 minutes, 4 °C) and determined the caspase 3 activity according to a standard curve.

### 2.7. Mitochondrial Respiration Monitoring

We used an XFe24 extracellular flux analyzer to detect the oxygen consumption rate (OCR) and extracellular acidification rate (ECAR) to monitor the mitochondrial oxidative phosphorylation reactions as previously described [17]. Briefly, we first pretreated the XFe sensor overnight at 37 °C in a CO_2_-free incubator. Then, we seeded the T47D and MCF-7 cells (1 × 10^5^/well) in a microplate and preincubated them for 1 h at 37 °C in a CO_2_-free incubator. Simultaneously, oligomycin, FCCP, and antimycin A were added to the loaded sensor cartridge. After a 30-min calibration time of the XFe sensor, with the preincubated sensor cartridge, the cell plate was loaded into the analyzer, and the ECAR of glycolytic capacity was measured and normalized by protein content.

### 2.8. Statistical Analysis

Data were presented as mean ± standard deviation. The differences between two groups were compared by Student's *t*-test. For multiple group comparison, we used one-way ANOVA followed by the Tukey *post hoc* test. A *P* value lower than 0.05 was considered statistically significant.

## 3. Results

### 3.1. Paeoniflorin Inhibits Proliferation but Does Not Induce Apoptosis in ER + BC Cells

**Figure 1:**
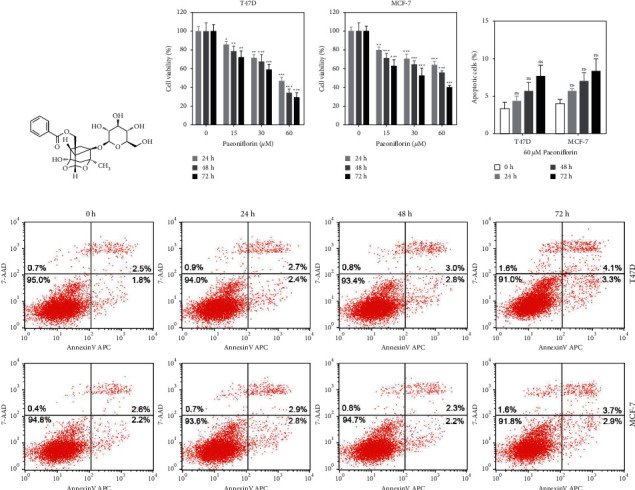
Cytotoxic effect of paeoniflorin on ER-positive breast cancer cells. (a) Molecular formula of paeoniflorin. (b, c) T47D and MCF-7 cells were treated with different concentrations of paeoniflorin (0, 15, 30, and 60 *μ*mol/L (*μ*M)) for different times (24, 48, and 72 hours), and the cell viability was determined using the CCK8 assay kit. (d) The apoptosis of T47D and MCF-7 cells were analyzed using a flow cytometer after stimulating with 60 *μ*M paeoniflorin for different times (24, 48, and 72 hours). (e) Representative flow cytometry scatter plot of cell apoptosis. Data were expressed as mean ± SD with 3 independent repetitions. ^ns^*P* > 0.05, ^*∗*^*P* < 0.05, ^*∗∗*^*P* < 0.01, and *P* < 0.001 vs without paeoniflorin treatment group.

Paeoniflorin, a kind of pinane monoterpene picroside (Figure 1(a)), is toxic to several malignant tumor cells. To assess the paeoniflorin toxicity on ER + BC cells, we treated the T47D and MCF-7 cells with different concentrations of paeoniflorin and measured the cell viability using a CCK8 kit 24, 48, and 72 hours after treatment. The results revealed that paeoniflorin significantly inhibited the T47D and MCF-7 proliferation (Figures 1(b) and 1(c)), and this effect was time and dose dependent. The inhibition of proliferation and induction of apoptosis caused the cell activity to be detected by CCK8 decreasing. Then, we detected the apoptosis of the T47D and MCF-7 cells after being treated with 60 *μ*M paeoniflorin for different times (0, 24, 48, and 72 hours). Compared with the control group (0 h. Cells that were treated without paeoniflorin), there was no significant change in the apoptosis rate of the T47D and MCF-7 cells in the paeoniflorin-treated groups (Figures 1(d) and 1(e)). Therefore, 60 *μ*M paeoniflorin within 72 hours of treatment did not induce the apoptosis of both cell lines but significantly inhibited their proliferation.

### 3.2. Paeoniflorin Enhances Tamoxifen-Induced Apoptosis in ER + BC Cells

**Figure 2:**
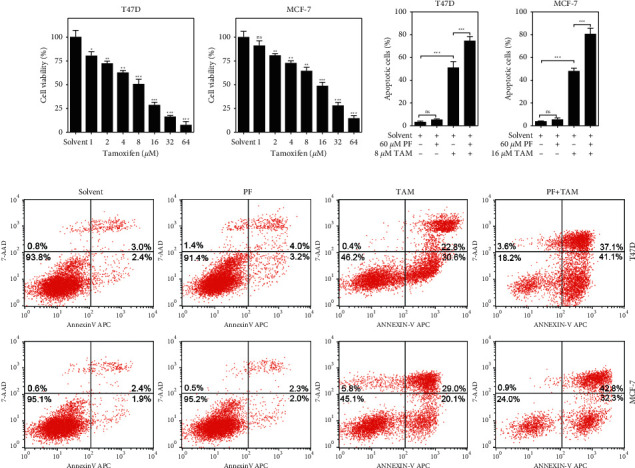
Paeoniflorin enhances tamoxifen-induced ER-positive breast cancer cells apoptosis. (a and b) T47D and MCF-7 cells were treated with different concentrations of tamoxifen (1, 2, 4, 8, 16, 32, and 64 *μ*M) for 48 hours, and the cell viability was determined using the CCK8 assay kit. Data were expressed as mean ± SD with 3 independent repetitions. ^ns^*P* > 0.05, ^*∗*^*P* < 0.05, ^*∗∗*^*P* < 0.01, and *P* < 0.001 vs solvent group. (c and d) T47D and MCF-7 cells were treated with or without PF (60 *μ*M) or TAM (8 *μ*M for T47D and 16 *μ*M for MCF-7) for 48 hours, and the apoptosis of T47D and MCF-7 cells were analyzed using a flow cytometer. Data were expressed as mean ± SD with 3 independent repetitions. ^ns^*P* > 0.05 and ^*∗∗∗*^*P* < 0.001 indicated difference between two groups. (e) Representative flow cytometry scatter plot of cell apoptosis. PF = paeoniflorin; TAM = tamoxifen.

Tamoxifen is a drug for the treatment of advanced ER-positive breast cancer. Herein, we treated the T47D and MCF-7 cells with different concentrations of tamoxifen for 48 hours and found that tamoxifen inhibited the T47D and MCF-7 (Figures 2(a) and 2(b)) cell activity in a dose-dependent manner. Furthermore, the activity of T47D cells treated with 8 *μ*M of tamoxifen for 48 hours was nearly half of that on the control group (Figure 2(a)), while the activity of the MCF-7 cells treated with 16 *μ*M of tamoxifen for 48 hours was about half of that found on the solvent control group (Figure 2(b)). In the following study, we chose the 8 *μ*M and 16 *μ*M concentrations of tamoxifen to treat the T47D and the MCF-7 cells, respectively. To evaluate the effect of paeoniflorin on the tamoxifen-induced toxicity in ER + breast cancer cells, we treated both cell lines with paeoniflorin or tamoxifen for 48 hours and determined the apoptosis levels by flow cytometry. The data revealed that the treatment with paeoniflorin alone did not induce T47D and MCF-7 cell apoptosis, while tamoxifen alone could significantly induce T47D and MCF-7 cell apoptosis (Figures 2(c)–2(e)). Notably, compared with the group treated with only tamoxifen, the T47D and MCF-7 cells' apoptosis rate was higher than that found in the paeoniflorin and tamoxifen combined treatment group, which suggests that paeoniflorin enhances tamoxifen-induced apoptosis in ER + BC cells.

**Figure 3:**
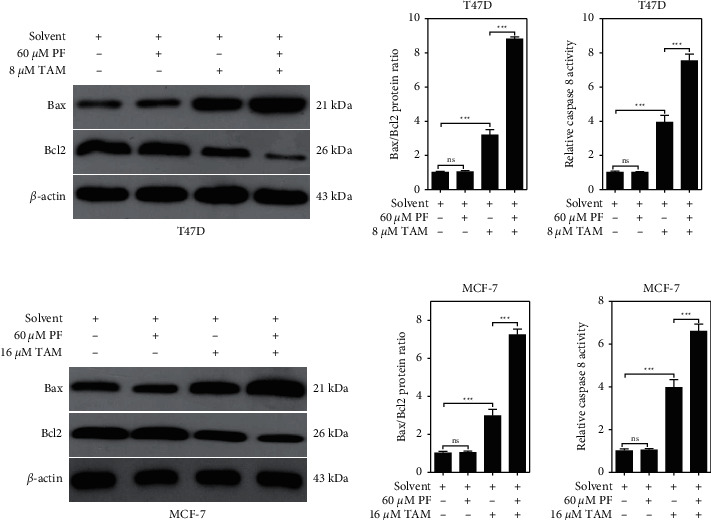
Effects of paeoniflorin on apoptosis-related protein expression after inducing with tamoxifen in ER-positive breast cancer cells. (a) Immunoblotting was used to detect the expression of Bax and Bcl2 in T47D cells after different treatments. (b) Statistically compare the ratio of BAX/Bcl2 protein expression in T47D cells of different groups. (c) The caspase 8 ELISA kit was used to detect the activity of caspase 8 in T47D cells of different groups. (d) Immunoblotting was used to detect the expression of Bax and Bcl2 in MCF-7 cells after different treatments. (e) Statistically compare the ratio of BAX/Bcl2 protein expression in MCF-7 cells of different groups. (f) The caspase 8 ELISA kit was used to detect the activity of caspase 8 in MCF-7 cells of different groups. Data were expressed as mean ± SD with 3 independent repetitions. ^ns^*P* > 0.05 and ^*∗∗∗*^*P* < 0.001 between two groups. PF = paeoniflorin; TAM = tamoxifen.

Apoptosis-related protein expression was also determined, and we found that the treatment with paeoniflorin alone did not significantly change the expression of the Bax/Bcl2 protein ratio (Figure 3(a) and 3(b)), nor the levels of caspase 8 activity in the T47D cells (Figure 3(c)). However, tamoxifen alone treatment could significantly increase the ratio of Bax/Bcl2 expression (Figures 3(a) and 3(b)) and the levels of caspase 8 activity in the T47D cells (Figure 3(c)). Notably, the expression of Bax/Bcl2 protein ratio and the levels of caspase 8 activity were all significantly increased in the combined paeoniflorin and tamoxifen treatment group when compared with the tamoxifen alone group. Similarly, this change in the Bax/Bcl2 protein expression ratio and the caspase 8 activity levels were also found in the MCF-7 cells (Figures 3(d)–3(f)).

### 3.3. Paeoniflorin Inhibits the Proliferation of ER + BC Cells by Increasing SIRT4

**Figure 4:**
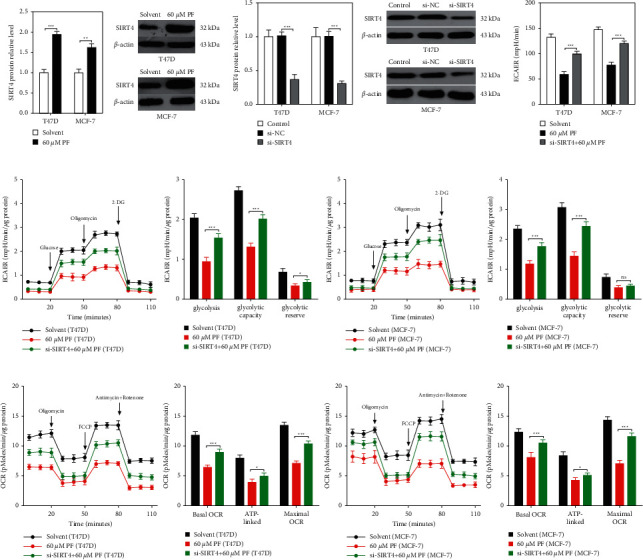
Paeoniflorin inhibits the proliferation of ER-positive breast cancer cells through SIRT4-mediated mitochondrial metabolism. (a) T47D and MCF-7 cells were treated with or without 60 *μ*M PF for 48 hours, and we harvested the cells to detect the expression of SIRT4 using western blot, and the representative proteins brand were showed in the right. (b) 48 hours after transfecting with specific small interfering RNA for SIRT4 (si-SIRT4), SIRT4 protein expression was analyzed by western blot in T47D and MCF-7 cells, and the representative proteins brand were showed in the right. (c) 48 hours after transfecting with si-SIRT4, T47D and MCF-7 cells were treated with or without 60 *μ*M PF for 48 hours, and the cell viability was determined using the CCK8 assay kit. 48 hours after transfecting with si-SIRT4, T47D, and MCF-7 cells were treated with or without 60 *μ*M PF for 48 hours, and we harvested the cells to detect the extracellular acidification rate (ECAR) (d) and oxygen consumption rate (OCR) (e) using a Seahorse XF24 extracellular flux analyzer. Data were expressed as mean ± SD with 3 independent repetitions. ^ns^*P* > 0.05, ^*∗*^*P* < 0.05, ^*∗∗*^*P* < 0.01, and ^*∗∗∗*^*P* < 0.001 between two groups.

Energy is necessary for cell proliferation and survival, and mitochondria are the central organelle for cell energy supply [18, 19]. SIRT4 has been implicated in regulating cancer cell toxicity by the modulation of glutamate dehydrogenase [15]. To study the mechanism of paeoniflorin toxicity on ER + BC cells, we detected the expression of the SIRT4 protein in T47D and MCF-7 cells after stimulating them with 60 *μ*M of paeoniflorin for 48 hours and found that paeoniflorin strongly increased the SIRT4 expression in both cell lines (Figure 4(a)). Next, we knocked down the expression of SIRT4 in the cells by transfecting them with a specific small interfering RNA for SIRT4 (si-SIRT4) to study the function of SIRT4 in the ER + BC cell toxicity regulation by paeoniflorin. Our data indicated a successful knockdown of the SIRT4 expression in the T47D and MCF-7 cells (Figure 4(b)). Macroscopically, the deletion of SIRT4 restored the decreased relative cell viability induced by paeoniflorin in the T47D and MCF-7 cells (Figure 4(c)). Then, a Seahorse XF24 extracellular flux analyzer was used to quantify the mitochondrial metabolism in the cells, and we found that the extracellular acidification rates (ECARs) (Figures 4(d) and 4(e)) were all significantly lower after the stimulation with 60 *μ*M of paeoniflorin. At the same time, the deletion of SIRT4 significantly increased the already decreased levels of ECAR after the stimulation with 60 *μ*M paeoniflorin. These results, taken together, indicate that paeoniflorin suppressed the ER + BC cell proliferation through SIRT4-mediated mitochondrial metabolism.

### 3.4. Paeoniflorin Increases SIRT4 Expression to Enhance Tamoxifen-Induced Apoptosis of ER + BC Cells

**Figure 5:**
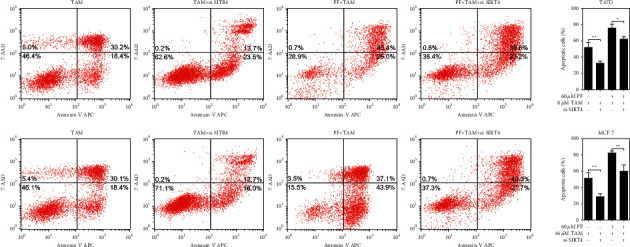
Deletion of SIRT4 increases paeoniflorin-enhanced tamoxifen-induced ER-positive breast cancer cell apoptosis. 48 hours after transfecting with si-SIRT4, T47D and MCF-7 cells were treated with or without PF (60 *μ*M) or TAM (8 *μ*M for T47D and 16 *μ*M for MCF-7) for 48 hours, and the cell viability was determined using the CCK8 assay kit. Data were expressed as mean ± SD with 3 independent repetitions. ^*∗*^*P* < 0.05, ^*∗∗*^*P* < 0.01, and ^*∗∗∗*^*P* < 0.001 between two groups. PF = paeoniflorin; TAM = tamoxifen.

**Figure 6:**
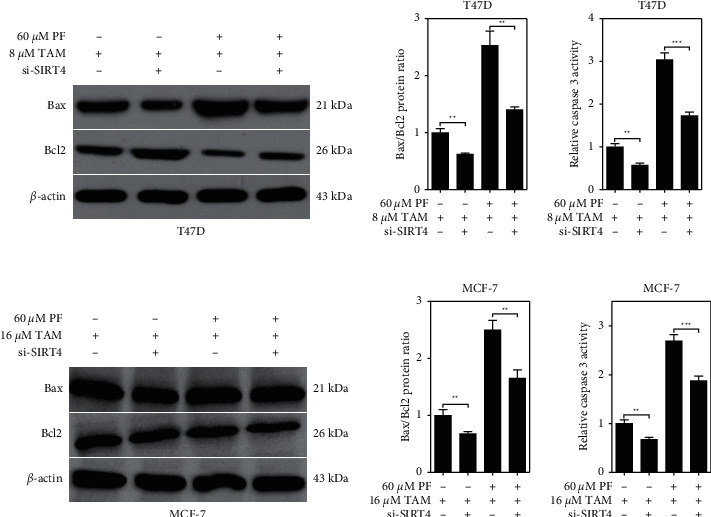
Paeoniflorin regulates apoptosis-related protein expression after being induced with tamoxifen, which is also related to SIRT4 expression. 48 hours after transfecting with si-SIRT4, T47D and MCF-7 cells were treated with or without PF (60 *μ*M) or TAM (8 *μ*M for T47D and 16 *μ*M for MCF-7) for 48 hours, and we harvested cells to detect the expression of Bax and Bcl2 protein expression using western blot and the activity of caspase 3 using the ELISA kit. Data were expressed as mean ± SD with 3 independent repetitions. ^ns^*P* > 0.05 and ^*∗∗∗*^*P* < 0.001 between two groups. PF = paeoniflorin; TAM = tamoxifen.

Tamoxifen kills breast cancer cells by inhibiting proliferation and promoting apoptosis. We explored the relation of paeoniflorin in tamoxifen-induced ER + BC cell apoptosis and the promotion of SIRT4 expression. To this, we detected the apoptosis levels in normal or SIRT4-knockdown T47D and MCF-7 cells after treating with tamoxifen alone or combined with paeoniflorin for 48 hours. Our results revealed that SIRT4 knockdown significantly decreased the apoptosis of T47D or MCF-7 cells after the treatment with tamoxifen alone or combined with paeoniflorin (Figure 5). Also, the cells were harvested to analyze apoptosis-related protein expression. We found that SIRT4 knockdown significantly decreased the Bax/Bcl2 expression ratio and the levels of caspase 3 activity in T47D or MCF-7 cells after the treatment with tamoxifen alone or combined with paeoniflorin (Figure 6).

### 3.5. Paeoniflorin Suppresses the SIRT4-Mediated STAT3 Activation in ER + BC Cells

**Figure 7:**
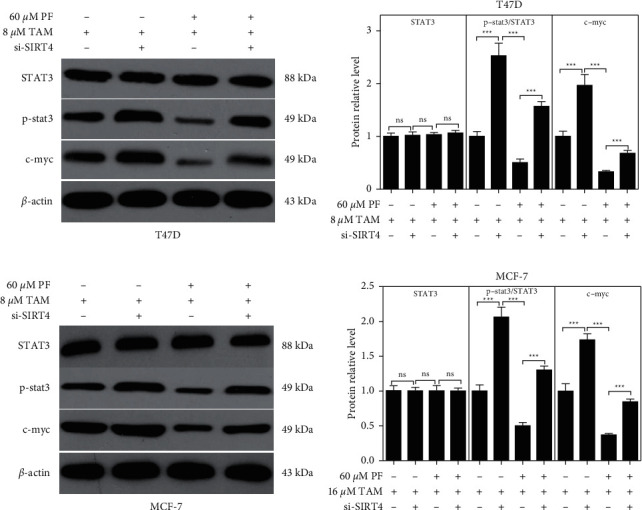
Deletion of SIRT4 increases the activation of STAT3 in ER-positive breast cancer cells. 48 hours after transfecting with si-SIRT4, T47D and MCF-7 cells were treated with or without PF (60 *μ*M) or TAM (8 *μ*M for T47D and 16 *μ*M for MCF-7) for 48 hours, and we harvested cells to detect the expression of c-myc, STAT3, and p-stat3 protein expression using western blot. Data were expressed as mean ± SD with 3 independent repetitions. ^ns^*P* > 0.05 and ^*∗∗∗*^*P* < 0.001 between two groups. PF = paeoniflorin; TAM = tamoxifen.

**Figure 8:**
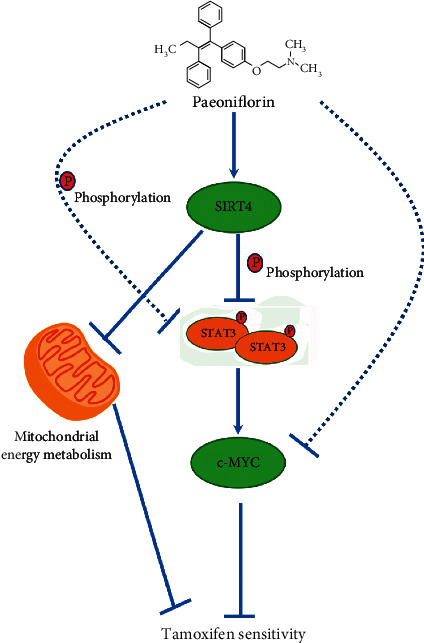
Paeoniflorin enhances the sensitivity of ER-positive breast cancer cells to tamoxifen through promoting SIRT4.

STAT3 is a signal transducer and activator of the transcription protein family, and its abnormal activation is related to the occurrence and development of several malignant tumors. SIRT4 enhances the sensitivity of ER + BC to tamoxifen by inhibiting STAT3 activation. Therefore, we evaluated the activation of STAT3 and found that tamoxifen treatment alone or combined with paeoniflorin did not change the expression of STAT3 protein, even the SIRT4 being knocked down. Simultaneously, the expression of phosphorylated STAT3 (p-stat3) had changed in T47D and MCF-7 cells (Figure 7). Besides, the expression of p-stat3/STAT3 was significantly decreased in T47D and MCF-7 cells stimulated with tamoxifen and paeoniflorin when compared with the tamoxifen-treated control group. Notably, the deletion of SIRT4 significantly increased the expression of p-stat3/STAT3 in T47D or MCF-7 cells treated with tamoxifen alone or combined with paeoniflorin (Figure 7). As targets of the STAT3 signaling pathway, c-myc gene amplifications confer tamoxifen resistance in ER + breast cancer [20]. Herein, we found that paeoniflorin could significantly decrease the expression of c-myc protein when the cell is stimulated with tamoxifen and the deletion of SIRT4 significantly increased the expression of c-myc in T47D or MCF-7 cells treated with tamoxifen alone or combined with paeoniflorin (Figure 7), suggesting that paeoniflorin improves the ER + tamoxifen sensitivity through the SIRT4-mediated STAT3 pathway (Figure 8).

## 4. Discussion

Breast cancer (BC) is one of the most common malignant tumors and the leading cause of tumor-related deaths in women, accounting for 11.7% of the new cancer patients each year worldwide [21, 22]. About 70–80% of breast cancer cases are positive for estrogen receptor expression, so endocrine therapy has become the primary and effective treatment for hormone-sensitive breast cancer [1, 2]. Tamoxifen is a commonly used drug for breast cancer endocrine therapy. As a selective ER regulator, tamoxifen can effectively block the stimulating effect of estrogen by binding with the ER and has noticeable therapeutic effects on patients with ER + BC [8, 23]. However, some significant problems must be addressed: side effects and drug resistance. Paeoniflorin is derived from natural plants and has low toxicity reported. At the same time, it presents recognized toxicity to many malignant tumors *in vivo* and *in vitro*, including breast cancer [24, 25]. Conversely, the effect of paeoniflorin on the sensitivity of ER-positive breast cancer cells to tamoxifen is still unclear. In the present study, we found that paeoniflorin inhibited the proliferation and enhanced the apoptosis of ER + breast cancer cells, both induced by tamoxifen. Cell lines are often used as *in vitro* tools to mimic specific types of *in vivo* systems. Several cell lines, including MCF-7 and T47D, have been widely used in breast cancer studies without investigating the particular cell line's characteristics [26]. Thus, our data revealed that this combined medication might reduce the tamoxifen dosage and side effects.

Studies reported that paeoniflorin inhibits tumor cell proliferation and metastasis and also induces apoptosis, but there are few studies on paeoniflorin regulating tumor cell sensitivity and drug resistance to chemotherapeutics. In a few reported studies, we found that paeoniflorin enhances the sensitivity of tumor cells to chemotherapeutics and reverses the drug resistance of tumor cells by regulating multidrug resistance genes [11, 12]. Previous studies have found the inhibitory effect of paeoniflorin on breast cancer [13, 14]. Moreover, our study provides direct evidence that paeoniflorin can enhance the toxicity of tamoxifen to ER + breast cancer cells. Other studies found that paeoniflorin could promote the SIRT4 expression and inhibit the mitochondrial metabolism of ER + BC cells. Additionally, the deletion of SIRT4 maintained the inhibitory effect of paeoniflorin on mitochondrial metabolism and cell proliferation. At the same time, the deletion of SIRT4 significantly decreased paeoniflorin-enhanced apoptosis induced by tamoxifen. Paeoniflorin has been found to regulate the biological characteristics of tumors through multiple targets [27, 28], and although the direct target of paeoniflorin has not been discovered yet, our data revealed that SIRT4 is a critical protein in the mechanism that paeoniflorin enhances the sensitivity of ER + breast cancer cells to tamoxifen.

SIRT4 can inhibit tumorigenesis by inhibiting glutamine metabolism in mitochondria [29, 30] and is considered a potential target for cancer treatment [31]. A previous study has found that SIRT4 is involved in a wide range of mitochondrial metabolic processes, including controlling apoptosis and regulating energy and redox metabolism [32]. Moreover, plant-derived compounds have been reported to induce metabolic reprogramming of breast cancer cells and enhance their sensitivity to chemotherapy drugs [33]. Glutamine is a key amino acid required for numerous intracellular processes, and its metabolite *α*-ketoglutarate can supplement the intermediate substances required for the tricarboxylic acid cycle, thereby meeting the energy needs of the rapid growth of cells [34, 35]. The survival and proliferation of tumor cells depend on glutamine, and SIRT4 can inhibit the glutamate dehydrogenase activity by regulating its ADP-ribosyltransferase cofactor, thereby inhibiting glutamine metabolism [36, 37] and finally preventing tumor cell proliferation and inducing apoptosis. In breast cancer, the loss of SIRT4 promotes the breast cancer stem cell's self-renewal [38] and is found related to the poor prognosis of patients with invasive breast cancer [38]. Notably, a new study found that the upregulation of SIR4 enhanced the sensitivity of ER + breast cancer cells to tamoxifen by inhibiting the activation of the STAT3 pathway [16]. In this study, we also found that paeoniflorin enhances the inhibitory effect of tamoxifen on STAT3 activation in ER + breast cancer cells.

As the confluence point of multiple oncogenic signal pathways, STAT3 is found to be abnormally activated at high frequency in malignant tumors and is a promising molecular target for cancer therapy [39, 40]. The overactivation of STAT3 is usually manifested as the level of tyrosine 705-phosphorylated STAT3 increased. Importantly, the level of tyrosine 705-phosphorylated STAT3 tamoxifen-resistant MCF-7/TAM cells is significantly higher than that in MCF-7 cells [41, 42]. In addition, we also found that paeoniflorin could significantly decrease the c-myc expression in cells stimulated with tamoxifen and the deletion of SIRT4 significantly increased the c-myc expression in T47D or MCF-7 cells treated with tamoxifen alone or combined with paeoniflorin. As a downstream gene of STAT3, c-myc was found to be related to the drug resistance of breast cancer cells [43, 44]. In conclusion, our data revealed that paeoniflorin promotes SIRT4 expression to enhance the sensitivity of ER-positive breast cancer cells to tamoxifen by suppressing STAT3 activation, suggesting that paeoniflorin could be used as a drug candidate for the treatment of ER + breast cancer. However, it is known that the same compound can have different effects on cells, animals, and humans. In the present study, we only checked the activity of paeoniflorin in ER + breast cancer cells, so research in animal models is very necessary in the future.

## Data Availability

The data used to support the findings of this study are available from the corresponding author upon request.
